# Characteristics and Antimicrobial Properties of Active Edible Films Based on Pectin and Nanochitosan

**DOI:** 10.3390/ijms21062224

**Published:** 2020-03-23

**Authors:** Thi Minh Phuong Ngo, Thanh Hoi Nguyen, Thi Mong Quyen Dang, Thi Xo Tran, Pornchai Rachtanapun

**Affiliations:** 1 Department of Chemical Technology and Environment, The University of Danang—University of Technology and Education, Danang 550000, Vietnam; ntmphuong@ute.udn.vn (T.M.P.N.); nthoi@ute.udn.vn (T.H.N.); 2 Department of Postharvest and Food Processing, Faculty of Food Technology, College of Food Industry, Danang 550000, Vietnam; mongquyen76@gmail.com; 3 Department of Chemical Engineering, The University of Danang—University of Science and Technology, Danang 550000, Vietnam; tranthixo@gmail.com; 4 Division of Packaging Technology, Faculty of Agro-Industry, Chiang Mai University, Chiang Mai 50100, Thailand; 5 Cluster of Agro Bio-Circular-Green Industry (Agro BCG), Chiang Mai University, Chiang Mai 50100, Thailand

**Keywords:** antimicrobial, active packaging, biopolymer, edible film, chitosan, nanochitosan, pectin

## Abstract

This study was aimed at creating new films and determine some functional packaging properties of pectin:nanochitosan films with ratios of pectin:nanochitosan (P:NSC) of 100:0; 75:25; 50:50; 25:75 and 0:100 (%*w*/*w*). The effects of the proportions of pectin:nanochitosan incorporation on the thickness, mechanical properties, water vapor permeability, water-solubility, and oxygen permeability were investigated. The microstructural studies were done using scanning electron microscopy (SEM). The interactions between pectin and nanochitosan were elucidated by Attenuated total reflectance-Fourier transform infrared (ATR-FTIR). The results showed that the blending of pectin with nanochitosan at proportions of 50:50 increased the tensile strength to 8.96 MPa, reduced the water solubility to 37.5%, water vapor permeability to 0.2052 g·mm/m^2^·day·kPa, and the oxygen permeability to 47.67 cc·mm/m^2^·day. The results of the contact angle test indicated that P:NCS films were hydrophobic, especially, pectin:nanochitosan films inhibited the growth of *Colletotrichum gloeosporioides, Saccharomyces cerevisiae*, *Aspergillus niger*, and *Escherichia coli*. So, P:NCS films with a proportion of 50:50 can be used as active films to extend the shelf life of food.

## 1. Introduction

Antimicrobial food packaging is a new type of active packaging incorporating natural antimicrobial compounds in order to reduce the food spoilage [1].

Pectin, composed of α-1,4-linked galacturonic acid, is a major component of plant cell walls and one of its attractive characteristics is its ability to form good films [2]. However, the pectin films have a poor water vapor barrier and a moderate oxygen barrier, therefore their utilization in food packaging is limited. To solve this problem, films based on pectin and nanochitosan are investigated in this study. Nanochitosan exhibits higher antimicrobial activities and barrier properties than chitosan. Hence, nanochitosan has the potential of becoming a powerful and safe natural antifungal agent which can improve the barrier properties and functionality of films and coatings [3].

Therefore, we have developed an inexpensive antimicrobial material which is composed of pectin and nanochitosan. Films formed between pectin chains and nanochitosans are stabilized by hydrogen links and electrostatic interactions [4]. The purpose of the present study was to evaluate some properties of active edible films based on pectin and nanochitosan.

In the present work, we aimed to prepare a pectin:nanochitosan polyelectrolyte complex to develop antimicrobial films. The developed films were characterized by mechanical properties, water vapor permeability, water contact angle, oxygen permeability, Fourier transform infrared (FTIR) spectroscopy and scanning electron microscopy (SEM). In addition, film-forming solutions were tested against four popular kinds of microorganisms which were *Escherichia coli*, *Aspergillus niger*, *Colletotrichum gloeosporioides*, and *Saccharomyces cerevisiae*.

## 2. Results and Discussion

### 2.1. Preparation and Characterization of Nanochitosan

**Figure 1:**
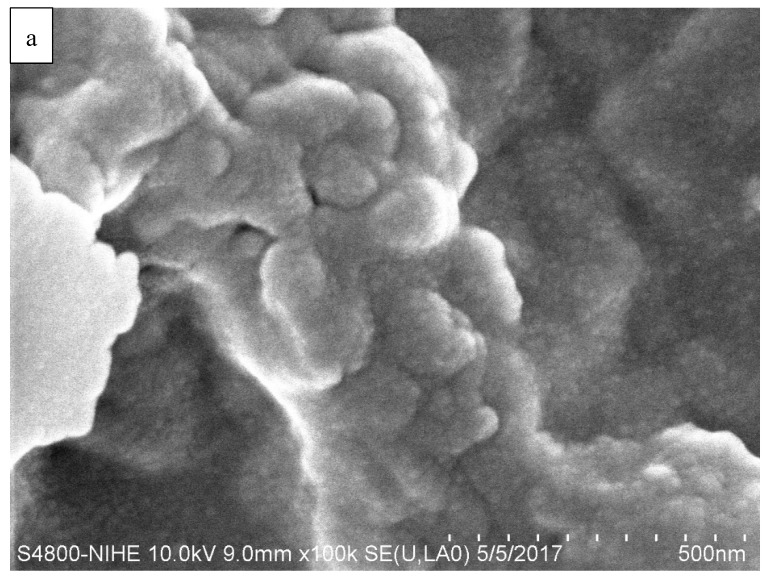

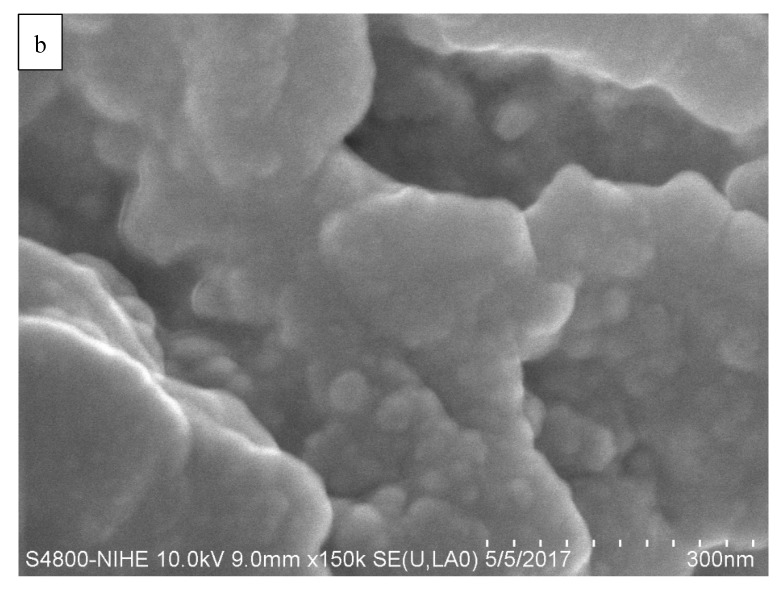
Scanning electron microscopy (SEM) micrograph of nanochitosan film. (**a**) 100,000× and (**b**) 150,000× magnification.

The nanochitosan was synthesized by the ionic methacrylic acid method according to de Moura [5]. The microstructure of the nanochitosan was studied by SEM at 100,000× and 150,000× magnification and the acquired micrograph for the nanochitosan film surface is shown in Figure 1. In this work, the particles are nearly spherical and the particle size diameters of the nanochitosan are less than 100 nm.

### 2.2. Thickness and Mechanical Properties of Films

Thickness values of the films are shown in Table 1. The pure pectin film was thicker than nanochitosan film solely. Increasing the content of nanochitosan from 25 to 50% affects the thickness of the composite films. However, on increasing the content of nanochitosan to 75%, the thickness of the nanochitosan film became higher than the pectin films. The decreasing thickness of the films is associated with electronic interaction between the positively charged nanochitosan and negatively charged pectin, caused by decreasing the charged groups of the polymer composite which resulted in a good aggregation between the polymer chains (due to the decrease of electronic repulsion). However, the presence of more nanochitosan caused an increase in the electronic repulsion which resulted in a reduction in the particle aggregation, hence an increased film thickness.

Mechanical properties (tensile strength (TS) and elongation (E)) of all the films are presented in Table 1. The tensile strength of the pectin films was 7.10 ± 0.22 MPa and increased to a maximum value of 10.84 ± 0.38 MPa when the nanochitosan concentration increased by 25%. However, further increases in the nanochitosan concentration (50 and 75%) decreased the TS of the films.

The enhanced TS and E values of P:NCS 75:25 and P:NCS 50:50 films can be explained by the higher number of interactions between the carboxylic groups of pectin and amine groups of nanochitosan. The influence of nanoparticles on the mechanical properties of films was investigated by Chang et al. and Parris et al. [6,7]. When nanochitosan particles were dispersed in the pectin matrix, the strong interaction between the nanoparticles and pectin may reduce the mobility and improve the tensile strength. Similarly, the results indicated that the P:NCS films presented a better elongation compared to the pectin films and nanochitosan films. In the pure pectin matrices and nanochitosan matrices, links are formed by hydrogen bonds and Van der Waals interactions. These weak interactions result in little flexibility and a brittle film. At nanochitosan concentration of 75%, the films became rougher and thicker and the interaction between pectin and nanochitosan is poor. As discussed above, adding nanochitosan at concentrations of 25 and 50% may improve the mechanical properties.

### 2.3. Contact Angle (CA) of the Films

The contact angles of the films are shown in Table 2.

Hydrophilicity can be investigated by the water contact angle of the film. The lower the contact angle, the more hydrophilic the film is [8]. As can be seen from Table 2, the initial contact angles of the pectin films and the nanochitosan films were 62.1° and 84.8°, respectively. These results suggest that the nanochitosan film was more hydrophobic than the pectin film. The addition of nanochitosan to the pectin films led to an improvement in the hydrophobicity of the film surface. As the ratio of nanochitosan increased from 25 to 75%, the contact angle increased from 89.3° to 97.1° for the P:NCS 75:25 and P:NCS 25:75 films, respectively, but there was no significant difference between the CA of the P:NCS 75:25 films and P:NCS 50:50 films.

The increase in CA of P:NCS films may be attributed to the ionic interactions formed between the nanochitosan and the pectin, reducing the hydrophilic groups. The higher the amount the hydrophobic acetyl groups in the nanochitosan, the higher the contact angle was. Moreover, the contact angle is also related to the roughness of the film surface.

### 2.4. Hydration Properties of the Films

Table 3 shows the solubility, swelling degree (SD), water vapor permeability (WVP), and water vapor transmission rate (WVTR) of the films. Pectin films exhibited high solubility when compared with the other films. Comparison of these values for films of different ratios of pectin and nanochitosan revealed a decrease in the solubility of P:NCS films when the proportion of nanochitosan was increased from 25 to 75%. The reduction of water solubility is a combined result of the optimum association between the NH_3_^+^ groups of nanochitosan and the COO^−^ groups of pectin.

Pectin, with a 43.48% degree of esterification (DE), contains a large number of carboxylic groups. The pKa of pectin and nanochitosan was found to be at pH 3.5 and 7.66, respectively. The pH of the solution in this study was 4.5, so the pectin chains were completely anionic and nanochitosan chains were fully protonated with free amino groups. The higher the number of cations in the nanochitosan, the better the electrostatic repulsion between their chains, leading to a higher swelling degree due to the solvation with more water molecules [4]. Similarly, the higher the anion content in the pectin, the higher the swelling degree of the pectin film is. As discussed above, the lowest SD of P:NCS 50:50 film can be explained in that ionic links were formed by the NH_3_^+^ groups of the nanochitosan and the COO^−^ groups of the pectin, therefore reducing the electrostatic repulsion between the chains.

When nanochitosan content was increased from 25 to 50% in the composite films, WVTR and WVP were decreased because the nanochitosan could be easily dispersed into the porous films. As a result, the more compact structure of the film formed more tortuous pathways for diffusion of water molecules and therefore, decreased the WVP and WVTR of the films. On the other hand, when nanochitosan was added into the pectin matrix, this reduced the number of carboxyl groups which decreased the WVTR and WVP values. At the nanochitosan concentration of 75%, the WVP and WVTR values increased because the surface of films became rougher and thicker due to the high concentration of nanochitosan, and there were more numerous and larger holes in the film matrices.

### 2.5. Moisture Sorption Isotherm of the Films

**Figure 2:**
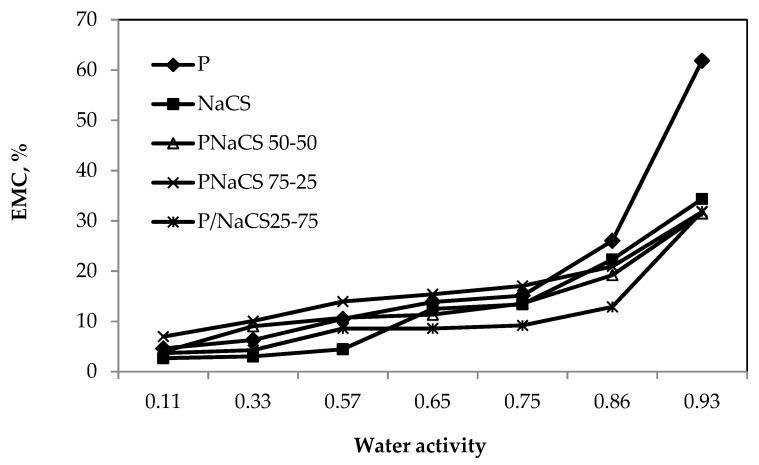
Moisture sorption isotherms of P:NCS films at 25 ± 0.5 °C.

Figure 2 shows the moisture sorption isotherms of the P:NCS films with various contents. The moisture absorption isotherm graphs of the P:NCS films with different contents have a characteristic type II sigmoidal shape.

Pectin films can have an uptake of up to 61.8% moisture when conditioned at 93% RH and 25 °C. Nanochitosan films, and all the P:NCS films with various ratios, have a moisture uptake of about <34.3% at 93% RH and 25 °C, a lower moisture uptake compared with the pectin films.

At each relative humidity, the equilibrium moisture content (EMC) of the P:NCS films decreased with increasing NCS concentrations, from 0 to 100%, with the maximum EMC obtained in a P and P:NCS 75:25 film. The P and P:NCS 75:25 films were more hydrophilic, therefore, they could absorb more moisture. There was a decrease in EMC value when increasing the nanochitosan concentration. It was noted that the film samples had water droplets on the surface at 99% RH and the EMC of films was over 200%. Therefore, these data points were omitted.

### 2.6. Color and Transparency of the Films

The color of films may affect the consumers’ acceptability of the products. The results of color assessments for the films are presented in Table 4. The a* value shows the color of the film changed from green to red as the a* value increased from a negative value to a positive value. An increase in the b* value indicated that the color of the film was becoming more yellow. The L* parameter was used to describe the contrast between dark and light with the range from 0 to 100. The chroma value shows the intensity of the color which changed from neutral to dull, and then to intense when the chroma increased from 0 to 50 and 100, respectively. The hue value shows the change of color from red-yellow to yellow, and then a yellowish-green color when the hue values increased from 49° to 90°, and 135°, respectively.

Table 4 shows the color values of the films at different ratios. Because NCS had a yellow color and the pectin film had a very slight yellow color, increasing the nanochitosan content significantly affected (*p* = 0.05) the a* and b* values of the film surface. Increasing the concentration of nanochitosan from 25 to 100% slightly increased the greenness a* values from −0.23 to 0.22. The color of the films changed to yellowish as indicated by an increase of the b* value from 2.57 for pectin films to 9.27 for the nanochitosan films, but the L* value of the P films and P:NCS films, with various proportions, were not significantly different.

### 2.7. Antimicrobial Action of Film-Forming Solutions

**Figure 3:**
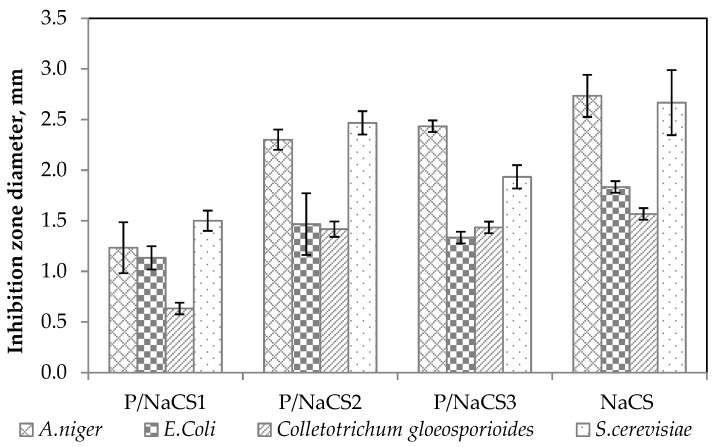
Antimicrobial activities of all films.

The antimicrobial action of the P:NCS solutions were tested against *E. coli*, *S. cerevisiae*, *A. niger*, and *C. gloeosporioides*. The inhibition zone diameter of the film-forming solutions against the growth of selected microorganisms is given in Figure 3. The antimicrobial activities of the P:NCS film-forming solution samples are predicted from the inhibition zone diameter measured in mm.

From the observed zone of inhibition values of all films presented in Figure 3, it was identified that film-forming solutions with nanochitosan had antibacterial activity towards four strains of microorganisms: *E. coli*, *S. cerevisiae*, *A. niger* and *C. gloeosporioides*. However, the pectin film did not have any inhibitory effect against the four tested microorganisms. Antimicrobial activity varied according to microorganism strains. These suspensions have been observed to act better on *Aspergillus niger* and *Saccharomyces cerevisiae* than on *Escherichia coli* and *Colletotrichum gloeosporioides*. The antimicrobial activity of NCS depends on its correlation with the physical characteristics of the nanoparticle size and surface charge of the microorganisms. These results demonstrate that nanochitosan exhibited antimicrobial activity due to the special characteristics of the nanoparticles such as small and compact particles, as well as high surface charge. The antimicrobial property of the film-forming solutions was improved with the increase in the concentration of NCS from 25 to 50%. The mechanism of antimicrobial activity of nanochitosan is proposed by two theories: First, nanochitosan with NH_3_^+^ groups will interact with the negatively charged surface of the microbial cell membrane so as to disrupt the functions of the cell membrane by breaking some components or by inhibiting the activity of cells, as a result, killing the microbial cells. Second, nanochitosan might be able to penetrate through the microbial cell membrane and bind to its DNA and as a result, prohibit the synthesis of the RNA, enzymes, and protein [9]. The reason for the reduced inhibition capacity of P:NCS 25:75 film compared with P:NCS 50:50 film could be caused by the aggregation and unequal dispersion of nanochitosan which decreased the contact between NCS and the microbial surface [10]. Antimicrobial activity of all the films also depends on the type of microorganism because of the different cellular structures and compositions of microorganisms, due to the mechanism of interaction between the -NH_3_+ groups of nanochitosan and the microbial structure. Importantly, chitosan nanoparticles exhibit potent bactericidal activity but do not show cytotoxicity on mammalian cells, according to Verma [11].

### 2.8. Oxygen Barrier Property of the Films

Oxygen is an important factor that causes oxidation which initiates several food changes which affects the shelf life of fruits [12]. The oxygen permeability (OP) and oxygen transmission rate (OTR) of all the films are presented in Table 5.

After investigating the solubility, WVP, WVTR, and antimicrobial activity of all the films, the P:NCS 50:50 film was found to be more suitable for application in food preservation than the P:NCS 75:25 film and the P:NCS 25:75 film. Hence, the P:NCS 50:50 film was selected for further investigation. OP and OTR of the films are shown in Table 5. The OTR of the P and NCS films were 671.0125 and 320.8 cc/m^2^·d, and the OP of P and NCS films were 1320.89 and 832.23 cc·mm/m^2^·d, indicating that these films were good oxygen barriers compared with low-density polyethylene and high-density polyethylene, with OTR values of 7000–8500, and 2300–3100 cc/m^2^·day, respectively. Generally, hydrophilic films show good oxygen barrier properties. A combination of pectin and nanochitosan helped decrease the OP of P:NCS 50:50 films. The improvement of the O2 barrier properties of the P:NCS 50:50 films was probably due to the formation of intermolecular hydrogen bonds between pectin chains NCS and hydrogen bonds between pectin and NCS, and the interaction of −NH_3_^+^ groups of NCS and –COO^−^ groups of pectin. The increased interaction resulted in a compact film with a low OP.

### 2.9. Fourier Transform Infrared (FTIR) Analysis of Pectin, Pectin:Nanochitosan and Nanochitosan Films

**Figure 4:**
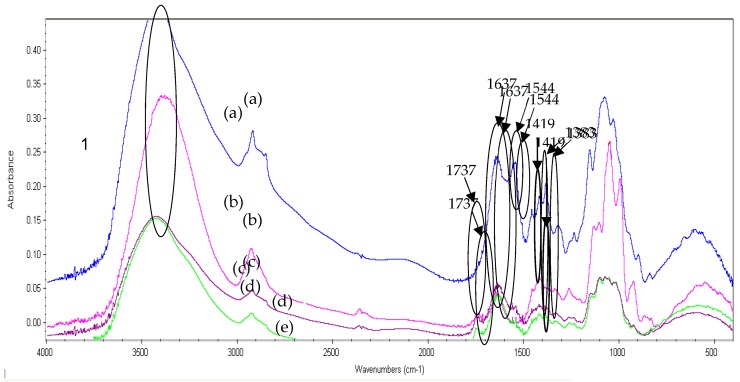
Fourier transform infrared (FTIR) spectra of nanochitosan film (**a**), P:NCS 50:50 film (**b**), P:NCS 75:25 film (**c**) and pectin film (**d**).

Figure 4 shows the spectra of the pectin films, the nanochitosan films, and the pectin-nanochitosan films. It is known that the peaks of the chitosan were related to the C=O stretching bands of amide I at 1635 cm^−1^ and to amide II at 1539 cm^−1^ [13]. These peaks slightly shifted to 1590 cm^−1^ in the nanochitosan. The absorption peak at 1590 cm^−1^ could be related to the bond formed between the NH^2^ groups of chitosan and methacrylic acid.

The peak of pectin is related to the C=O stretching in the ester form at 1737 cm^−1^, the C=O stretching at 1637 cm^−1^, and the COO^−^ symmetric stretching at 1417 cm^−1^ [14]. The peak of nanochitosan is related to the N-H of amide I stretching at 1630 cm^−1^, N-H of amide II stretching at 1544 cm^−1^, and –NH_3_^+^ symmetric stretching at 1383 cm^−1^.

For the spectra of P:NCS films, the main changes were in the range of 1500–1700 cm^−1^ corresponding to the interaction of amine groups on nanochitosan and the carboxylic groups of pectin. After reacting, the vibrational band corresponding to the primary amino groups at 1544 cm^−1^ disappeared while bands at 1419 and 1383 cm^−1^ were formed, indicating that there are interactions between pectin and nanochitosan. The increase in tensile strength values (Table 1) upon NCS addition illustrates pectin and nanochitosan interactions. NaCS addition decreases the number of OH groups in the pectin matrix. As a result, nanochitosan interactions may reduce the length of the pathway for water and gas migration, which may be due to the decreased permeance. These results confirmed that a reaction had occurred between pectin and NCS.

### 2.10. Scanning Electron Microscopy (SEM)

**Figure 5:**
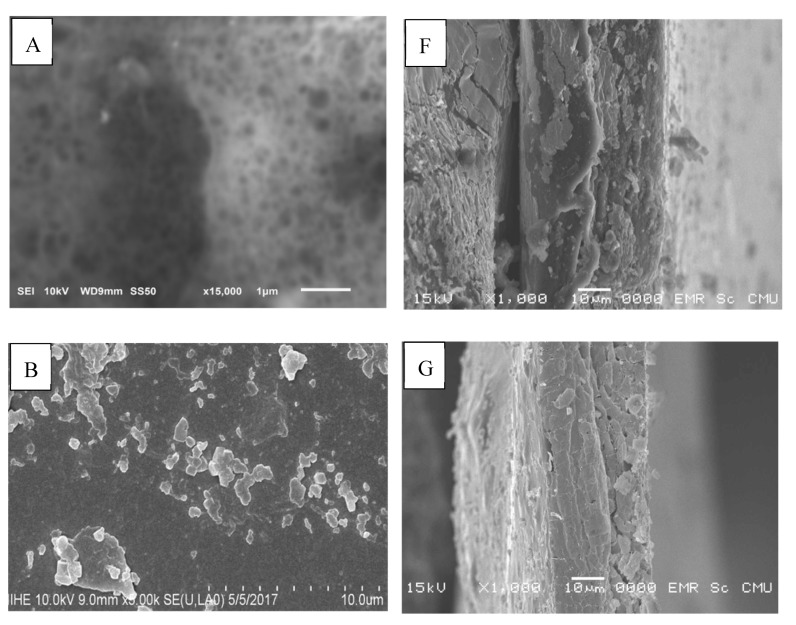

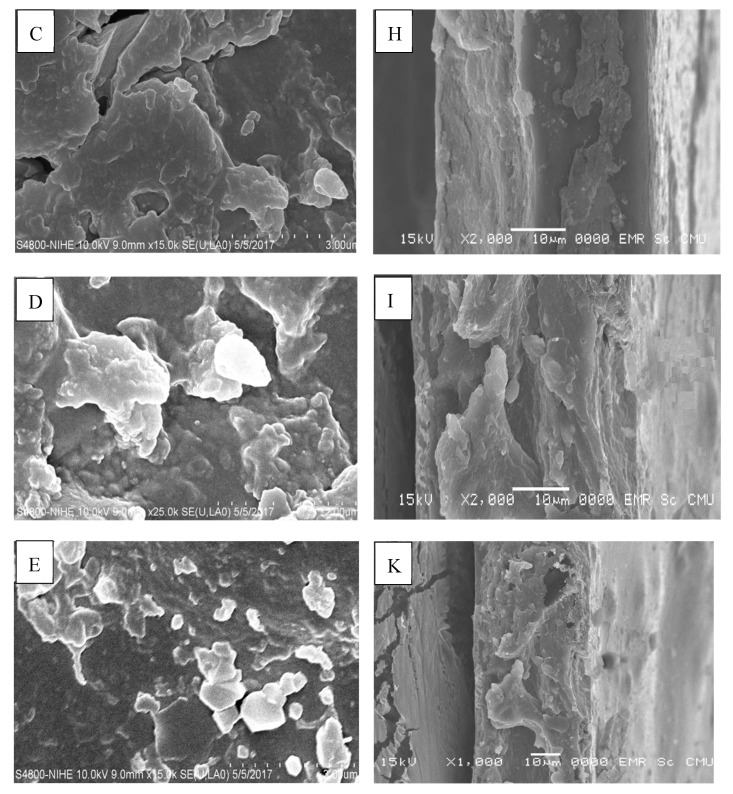
SEM images of surface of P, P:NCS 75:25, P:NCS 50:50, P:NCS 25:75, NCS films (**A**–**E**, respectively) and SEM images of cross-section of P, P:NCS 75:25, P:NCS 50:50, P:NCS 25:75, NCS films (**F**–**K**, respectively).

The SEM images of the surface and cross-section of P, P:NCS 75:25, P:NCS 50:50, P:NCS 25:75, and NCS films are shown in Figure 5.

The pectin film presented a smooth and uniform structure with only a few small solid particles on the surface. The nanochitosan films had rougher surfaces and contained some holes, as seen throughout the images of the surface and cross-section. These holes were probably the result of defects formed in the films caused by the repulsion force of positively charged groups in the nanochitosan. The surface of P:NCS 50:50 film was smoother than NCS film’s, which indicates good compatibility between pectin and nanochitosan. The microstructure of the P:NCS film presents small pores across the surface, thus showing the low water vapor permeability, oxygen permeability, and high tensile strength. Moreover, interactions between NCS and P made the surface of the films more hydrophobic, which relates to a lower WVP and water solubility.

## 3. Materials and Methods

### 3.1. Materials

Chitosan (CS) (MW = 70 kDa, degree of deacetylation of 90%) was purchased from Vietnamese Chitosan Co., Ltd. Methacrylic acid (MAA), potassium persulfate (K_2_S_2_O_8_), and acetic acid (CH3COOH) were purchased from Merck, Germany. Glycerol and CaCl2 were purchased from Xilong, China and BHI medium was purchased from Sigma–Aldrich, Saint Louis, MO, USA.

The pectin (P) was extracted from Tiliacora triandra using a heating method [15]. The degree of esterification of pectin was 48.36 and its molecular weight was 111.1 kDa.

Nanochitosan (NCS) were prepared by polymerizing methacrylic acid (MAA) in chitosan (CS) solution according to the method described by Nguyet and de Moura [5,16,17]. Firstly, chitosan was dissolved in a 0.5% MMA solution for 12 h. The chitosan concentration used in the synthesis was 0.8 *w*/*w*%. K_2_S_2_O_8,_ with a concentration of 0.6 mmol was then added to the chitosan—methacrylic solution at 70°C for 1 h under stirring. After that, the mixture was cooled at 4 °C. The suspension was centrifuged at 4000 r/min for 30 min and a suspension was obtained [5]. Chitosan nanoparticles were twice washed in distilled water, producing a chitosan nanoparticles suspension (NCS).

### 3.2. Film Preparation

The pectin:nanochitosan films were prepared as follows: 2 g pectin was dissolved in 98 g distilled water and homogenized at 60 °C. Glycerol was used as a plasticizer at 50% (%*w*/*w* of polymer). After complete dissolution, the pH was adjusted to 4.5 measured by a pH meter. Then, 2% nanochitosan suspension was added slowly into the pectin solution. The mixture was vigorously stirred for an hour at room temperature. The mixture was stored at 4 °C without mixing for a day to remove air bubbles. The final solution was poured onto a series of molds (size 15 cm × 15 cm). Films were dried, peeled off, and conditioned at 53 ± 1% RH, and 25 ± 1°C for 5 days before testing.

Five films were prepared with the ratios of pectin:nanochitosan (P:NCS) as follows: 100:0, 75:25, 50:50, 25:75 and 0:100.

### 3.3. Film Thickness

Film thickness was determined by a thickness gauge (Model PCM 137, No.2046S, Tokyo, Japan) with 0.01 mm accuracy. Ten random positions on the film sample were used to measure the thickness. The measurements were repeated in triplicate.

### 3.4. Film Solubility

The water solubility of the film was measured as the percentage of dry matter of the film solubilized in water after 24 h according to Gontard, et al. [18]. Tests were performed in triplicate.

### 3.5. Swelling Degree (SD)

Films with a dimension of 25 mm × 25 mm were weighed and immersed in distilled water at 25^°^C for 50 min. Water was then removed from the films by placing on dry filter papers and the films were re-weighed. Tests were repeated in triplicate [19]. The swelling degree was determined using Equation (1):

(1) $$SD=\frac{m_{f-}m_{i}}{m_{i}}\;\times \;100,\;\%$$

Where *m_f_* and *m_i_* are the weights of the swollen and initial samples, respectively.

### 3.6. Water Vapor Permeability (WVP) and Water Vapor Transmission rate (WVTR)

The WVP of the films was measured gravimetrically at 25 °C (± 1 °C) according to ASTM method E96 (2000). The preparation process was described in detail by Rachtanapun [20]. Tests were performed in triplicate. The WVP was calculated using Equation (2):

(2) $$MVP=\frac{w.x}{t.A.P_{0}.\left(RH_{1}-RH_{2}\right)}\;$$

Where: *w*/*t* is the constant rate of weight change when a straight line adequately fits the weight change versus time plot by linear regression with *R^2^* > 0.99, *x* is the thickness of film (mm), *A* is the test area (23.76 cm^2^), *P_o_* is the partial pressure of the water vapor at 25 °*C* (3.159 kPa), and (*RH_1_−RH_2_*) is the relative humidity difference between the two sides of the film [21].

The WVTR of the films was calculated from the slope of the straight line (g/day) divided by the transfer area (m^2^).

### 3.7. Moisture Sorption Isotherm

Moisture sorption was determined according to the method reported by Ngo et al. [21]. The equilibrium moisture content (*EMC*) (g water/100 g dry solid) of the samples at each water activity was determined using Equation (3) [22]:

(3) $$EMC=\frac{W_{e}}{W_{i}}\left(M_{i}+1\right)-1$$

Where *EMC* is the equilibrium moisture content (g water/100 g dry solid), *W_e_* is the equilibrium weight of the film (g), *W_i_* is the initial weight of the film (g), and *M_i_* is the initial moisture content of the film (g/g) [21]. Tests were performed in triplicate.

### 3.8. Mechanical Properties

Mechanical properties were directly measured via the tensile properties and % elongation of the films by a Universal Testing Machine H1KS using the ASTM D882-10 method [8]. The films were cut into rectangles of 150 mm × 25 mm. The initial grip separation was 125 mm and the rate of grip separation was 12.5 mm/min. The parameters determined were maximum load at break (MPa), and extension of length at rupture (%). The tensile strength (TS) and percentage of elongation (%E) for each film were calculated using the following Equations (4) and (5) [23]:

(4) $$TS=\frac{Maximum\;load\;at\;break}{Transverse\;section\;area},\;N$$

(5) $$\%E=\frac{Extension\;of\;length\;at\;rupture}{Initial\;length}\times 100,\;\%$$

### 3.9. Color Measurement

Color (L*, a*, b*, chroma, and hue) of films were measured using the Hunter lab color meter Color Quest XE (The Color Management Company, Reston, VA, USA) calibrated with a white and a black tile. Tests were repeated in triplicate for each treatment [24].

### 3.10. Water Contact Angle

The water contact angle was determined by drop shape analysis (DSA30E, Krüss Co. Ltd., Germany). The room temperature was set at 25.0 ± 0.1 °C. The water droplet with a volume of 10.0 ± 0.5 µL was dropped on a solid surface and images were taken every 6 s for 1 min [25].

### 3.11. Oxygen Permeability

The oxygen permeability (*OP*) of the film was determined according to the ASTM D-3985 method using the OX-TRAN model@ 2/21 system (Minneapolis, USA) [26]. *OP* was determined using Equation (6):

(6) $$OP=\frac{OTR.l}{\Delta P}$$

Where *OP* (cc.mm/m^2^.day) and *OTR* (cc/m^2^·day), (*ΔP*) is the difference in oxygen partial pressure between the two sides of the film, and l is the thickness of the film.

### 3.12. Fourier transform infrared (FTIR) spectroscopy

P, NCS, and P/NCS films were characterized by Fourier transform (FTIR) spectrophotometer (Thermo Nicolet 6700, Waltham, MA, USA). The FTIR spectra were recorded in absorbance mode at a wavelength range of 500 cm^−1^ to 4000 cm^−1^ and with a resolution of 4 cm^−1^.

### 3.13. Scanning Electron Microscopy (SEM)

The surface and cross section of the P, NCS, and P/NCS films were investigated by scanning electron microscopy (JEOL, JSM-5910LV, Tokyo, Japan). Cross-sectional samples were dipped in liquid nitrogen and then coated with gold under vacuum. The surface and cross-sectional morphologies of the film were recorded with over 1000 × magnification [24].

### 3.14. Antimicrobial Action of Film-Forming Solutions

The antimicrobial activity of all film-forming solutions was tested against contaminating microorganisms typically found in food and fruit (*Saccharomyces cerevisiae, Escherichia coli*, *Colletotrichum gloeosporioides, Aspergillus niger*) using the agar well diffusion method. This method was described in detail by Ngo [21].

### 3.15. Statistical Analysis

Minitab 16 software (Version 16.2.3.0, Minitab Statistical Software, Bac Tu Liem, HN, Vietnam) was used to analyze the results in this study. Comparisons among multiple groups were evaluated by one-way analysis of variance (ANOVA).

## 4. Conclusions 

In this work, the new films composed of pectin (P) and nanochitosan (NCS) were obtained. The blending of pectin with nanochitosan led to an improvement of mechanical properties, barrier properties, and hydration properties. Moisture sorption isotherms indicated the hydrophobic character of the P:NCS films, and the pectin:nanochitosan films, especially, inhibited the growth of *S. cerevisiae, A. niger, C.gloeosporioides* and *E. coli*. The ATR-FTIR spectra and SEM images for P:NCS 50:50 film indicated good interaction of film-forming components. These characterizations show that the pectin and nanochitosan films are a new approach for the development of active packaging for extending the shelf life of food products.

## Figures and Tables

**Table 1:** Thickness and mechanical properties of films.

Films	Thickness, µm	Tensile Strength, MPa	Elongation, %
P:NCS 100:0	54.0 ± 2.50 ^b^	7.10 ± 0.22 ^b^	7.17 ± 0.35 ^b^
P:NCS 75:25	46.5 ± 2.05 ^c^	10.84 ± 0.38 ^a^	17.22 ± 0.9 ^a^
P:NCS 50:50	47.7 ± 1. 8 ^c^	8.96 ± 0.19 ^b^	10.60 ± 0.29 ^b^
P:NCS 25:75	61.2 ± 2.46 ^a^	2.86 ± 0.09 ^c^	8.65 ± 0.42 ^b^
P:NCS 0:100	43.0 ± 1.46 ^c^	3.57 ± 0.15 ^c^	10.52 ± 0.43 ^b^

Note: Alphabets (a, b and c) in a column represent significant differences (*p* ≤ 0.05).

**Table 2:** The contact angle of films.

Films	Contact angle, °		Image	
	Initial CA	After 12 s	Initial CA	After 12 s
P:NCS100:0	62.1 ± 2.12 ^c^	45.20 ± 2.96 ^d^	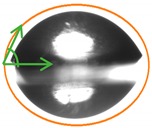	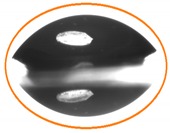
P:NCS 75:25	89.3 ± 6.86 ^ab^	82.60 ± 6.85 ^b^	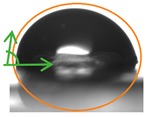	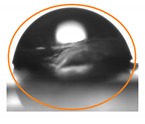
P:NCS 50:50	95.4 ± 10.74 ^ab^	86.23 ± 3.01 ^ab^	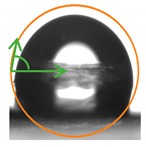	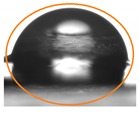
P:NCS 25:75	97.1 ± 2.9 ^a^	92.5 ± 3.8 ^a^	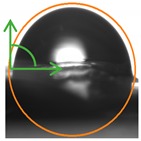	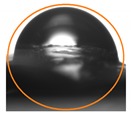
P:NCS0:100	84.80 ± 1.75 ^b^	73.37 ± 1.90 ^c^	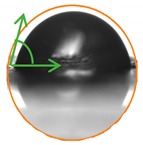	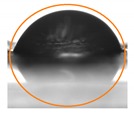

Note: Alphabets (a, b, c and d) in a column represent significant differences (*p* ≤ 0.05).

**Table 3:** Hydration properties of the films.

Films	Solubility, %	SD, 50 m %	WVP, g·mm/m^2^·day·kPa	WVTR, g/m^2^·d
P: 100:0	100 ± 0 ^a^	−100 ± 0 ^d^	1.33 ± 0.0285 ^a^	38.77 ± 0.83 ^a^
P:NCS 75:25	45.65 ± 1.69 ^b^	249.45 ± 12.73 ^b^	0.2699 ± 0.0089 ^c^	9.91 ± 0.33 ^c^
P:NCS 50:50	37.5 ± 1.69 ^c^	117.8 ± 8.77 ^c^	0.2052 ± 0.0083 ^d^	8.10 ± 0.33 ^d^
P:NCS 25:75	11.11 ± 1.13 ^d^	230.98 ± 11.38 ^b^	0.5922 ± 0.0204 ^b^	17.26 ± 0.60 ^b^
P:NCS 0:100	37.84 ± 1.98 ^c^	443.15 ± 21.28 ^a^	0.1755 ± 0.0085 ^d^	9.24 ± 0.45 ^cd^

Note: Alphabets (a, b, c and d) in a column represent significant differences (*p* ≤ 0.05).

**Table 4:** Color of films.

Films	Parameters				
	L^*^	a^*^	b^*^	Chroma	Hue
P:NCS 100:0	96.24 ± 0.31 ^a^	0.14 ± 0.01 ^b^	2.57 ± 0.12 ^d^	2.57 ± 0.11 ^d^	86.81 ± 0.5 ^b^
P:NCS 75:25	95.76 ± 0.2 ^a^	-0.23 ± 0.01 ^d^	5.98 ± 0.31 ^c^	5.99 ± 0.25 ^c^	87.09 ± 0.73 ^b^
P:NCS 50:50	95.69 ± 0.31 ^a^	-0.13 ± 0.02 ^c^	6.59 ± 0.63 ^c^	6.60 ± 0.63 ^c^	88.12 ± 0.11 ^ab^
P:NCS 25:75	95.48 ± 0.25 ^a^	0.10 ± 0.01 ^b^	7.49 ± 0.32 ^b^	7.49 ± 0.32 ^b^	89.25 ± 0.26 ^a^
P:NCS 0:100	94.51 ± 0.18 ^b^	0.22 ± 0.01 ^a^	9.27 ± 0.43 ^a^	9.28 ± 0.42 ^a^	88.62 ± 0.35 ^a^

Note: Alphabets (a, b, c and d) in a column represent significant differences (*p* ≤ 0.05).

**Table 5:** Oxygen permeability (OP) and oxygen transmission rate (OTR).

Films	OP, cc·mm/m^2^·d	OTR, cc/m^2^·d
P:NCS 100:0	1320.89 ± 88.29 ^a^	671.01 ± 47.11 ^a^
P:NCS 75:25	836.89 ± 56.12 ^c^	350.54 ± 18.99 ^bc^
P:NCS 50:50	47.67 ± 5.11 ^d^	18.63 ± 2.17 ^d^
P:NCS 25:75	898.75 ± 61.44 ^b^	409.03 ± 35.63 ^b^
P:NCS 0:100	832.23 ± 49.89 ^c^	320.8 ± 25.88 ^c^

Note: Alphabets (a, b and c) in a column represent significant differences (*p* ≤ 0.05).

## Abbreviations

NCS	Nanochitosan
P	Pectin
SEM	Scanning electron microscope
FTIR	Fourier Transform infrared
MAA	Methacrylic acid
CS	Chitosan
